# In Vitro Assessment of Eight Selected Indigenous Fungal Isolates Tolerance to Various Abiotic Stresses and their Effects on Seed Germination

**DOI:** 10.1007/s00284-023-03507-6

**Published:** 2023-10-24

**Authors:** Mukondeleli N. Ramatsitsi, Mbokota C. Khosa, Chuene V. Mashamaite, Khosi Ramachela

**Affiliations:** 1 School of Agricultural Sciences, North-West University, Private Bag X2046, Mahikeng, 2745 South Africa; 2 Agricultural Research Council-Tropical and Subtropical Crops, Private Bag X11208, Mbombela, 1200 South Africa; 3 Department of Agronomy, University of Fort Hare, Private Bag X 1314, Alice, 5700 South Africa; 4 Food Security and Safety Niche Area, Crop Science Department, North-West University, Private Bag X2046, Mahikeng, 2745 South Africa

## Abstract

Fungal bio-control agents (BCA) can minimize use of agro-chemicals while increasing plant productivity and tolerance to biotic-abiotic stressors. Ideally, BCA should tolerate varying environmental conditions they are introduced into, to successfully dominate and protect plants from stressors. However, BCA are living micro-organisms, their survival and efficacy can be impeded by extreme conditions. The current study aimed at evaluating whether indigenous fungal isolates, viz, *Aspergillus flavus*, *A*. *terreus*, *Penicillium* sp. AL-38 IRH-2012b, *Talaromyces minioluteus*, *T*. *purpureogenus*, *T*. *sayulitensis*, *Trichoderma ghanense* and *T*. *viride* can tolerate different levels of salinity, pH, nutrient and temperature. Certain fungal species are pests with potential of destroying many crops; the pathogenic effects of the aforementioned fungal isolates were further assessed on different crops’ seeds. The results showed that, although being indigenous, *Aspergillus*, *T*. *sayulitensis* and *T*. *ghanense* failed to thrive in high salinity and pH. While *Penicillium* sp. AL-38 IRH-2012b failed to thrive under reduced nutrient level and all fungal isolates failed to grow at 10–20 °C. Furthermore, it was noted species within the same genus could affect crops in both favorable and unfavorable ways. The study demonstrated that the selected indigenous fungal isolates can tolerate different abiotic conditions and have potential to improve seed germination and seedling growth.

**Supplementary Information:**

The online version contains supplementary material available at 10.1007/s00284-023-03507-6.

## Introduction

Due to their importance in human diet, leafy vegetables and grains are globally cultivated and consumed as sources of essential nutrients and fiber [1, 2]. These crops are grown either in open fields or intensively managed environmentally controlled settings. However, soil-borne pathogens represent a severe threat to optimal crop production [3, 4]. Consequently, low crop production could impede farmers and communities from contributing to 2030 Sustainable Development Goals (SDGs 1–3) of No Poverty, Zero Hunger, and Good Health and Well-being (United Nations 2020). Moreover, as consumers demand for year-round production of fresh produce with little to no chemical residues, growers face even more difficulties managing pathogens. Currently, researchers are focusing on studying microbes that are both safe for humans and the environment [5, 6].

In South Africa (SA), indigenous fungal isolates have demonstrated tremendous potential as bio-control agents (BCA) against plant pathogens [7, 8]. However, abiotic conditions like temperature have an impact on BCA effectiveness [9]. *Aspergillus*, *Penicillium*, *Talaromyces* and *Trichoderma* species have been the subject of extensive research in plant pathogen management [10, 11]. Several studies have attributed fungal BCA capacity for bio-control to the production of antimicrobial substances, lytic enzymes, pathogenesis-related proteins, phenolic secondary metabolites, as well as their ability to outcompete other root pathogens and inducing systemic resistance in plants [12, 13].

Soil-borne plant diseases caused by soil-borne pathogen are brought about by ecosystem imbalances such as changes in planting conditions and local environment. As a result, abiotic stress is a crucial element that significantly affects agricultural soil fertility and productivity [14]. Abiotically stressed soils that are acidic with high salinity levels and nutrient-deficiency were shown to benefit very slightly from the use of BCA; regardless of whether bio-agents have bio-control or bio-fertilizer qualities [15]. Therefore, it is imperative to determine abiotic stress tolerance in beneficial soil fungi. This is because fungal BCA that have evolved to thrive in harsh conditions, such as high salinity or relatively high temperatures, may in turn have an impact on their symbiotic hosts’ ability to adapt to a comparable spectrum of situations.

It is generally recognized that two key characteristics must be present or improved when choosing acceptable fungal BCA for use as biocides: (i) high virulence against the intended pests and (ii) environmental stress tolerance [15, 16]. Despite the fact that these BCA have been studied for their potential to parasitize plant pathogens [5, 6], little research focus has been placed on determining how well these isolates can withstand abiotic stress and/or recover from it. Additionally, there are several species of fungi that are pests in and of themselves, destroying unfathomable amounts of crops, forests, stored goods and structures, while also harming both human and animal health [17, 18]. For these reasons, determining the unintended consequences of using indigenous fungi as BCA is necessary before developing and using them.

Considering that field studies would require handling a wide range of complex soil types, an in vitro study was therefore formulated. The objective of the study was to carry out in vitro assessment of tolerance level of selected indigenous fungal isolates to various abiotic stresses i.e., salinity, pH, nutrient status and temperature. The study also looked at fungal isolates’ biosafety on seeds.

## Materials and Methods

### Study Area

The study was conducted at North-West University, Mahikeng, SA, during winter season (June–July 2022). The climate of the area is classified as subtropical, is characterized by an average temperature of 5 °C in winter and 32 °C in summer and summer rainfall range between 1100 and 1600 mm/year.

### Fungal isolates

Fifty soil samples around plant roots, with no specific target plants, were collected using a garden trowel at North-West University, agricultural research farm, Molelwane, Mmabatho (latitude of 25.810 S and longitude of 25.630 E). The soil samples were transported to the laboratory in sterile polythene zip-lock bags and investigated for the presence of fungi. For fungal isolation, soil serial dilution plate method was used [19]. Serial dilutions were made from 10 g in 100 mL soil suspension with sub-subsamples being cultured on Petri dishes containing potato dextrose agar (PDA) media (Biolab, Lawrenceville, Georgia, USA) (39 mg L^−1^) treated with Streptomycin (0.5 g L^−1^) (Mast Diagnostics, UK). The PDA media was specifically used to enable isolation of both endophytic and free-living fungi that have direct and indirect soil ecological functions such as antibiosis and mycoparasitism. The Petri dishes were then incubated at 25 ± 2 °C and observed every two days for re-isolation of pure cultures [20]. Individual fungal colonies were each isolated and purified. Seven-day-old pure culture mycelia of respective fungal isolates were collected from the colony edges and microscopically examined. The hyphae and spore color, size and shape were used to identify the different species [21]. The morphological identification was supplemented by molecular identification using sequencing of internal transcribed spacer (ITS) gene region (with ITS1 and ITS2 sub-regions), and their identities were confirmed by taxonomist, Inqaba Laboratory, Tshwane, Gauteng Province, SA.

### Soil Tests

The same soil samples used for serial dilutions were air-dried and sieved through a 2-mm sieve. Soil pH, electrical conductivity (EC), soil texture and organic compost (OC) were determined. Soil pH was tested using the Bouyoucos hydrometer technique [22], and EC was measured following Richard [23] procedure. Soil texture was determined using standard glass/calomel electrodes in a 1:2.5 w/v soil–water suspension [24]. Soil OC was tested following a method by Walkley [25].

### Tolerance of Indigenous Fungal Isolates To Extreme Environmental Conditions

To investigate the abiotic stress tolerance of the selected indigenous isolates, a concentration gradient culture was established following a modified procedure by Yu et al. [26]. For matching saline-alkali land, NaCl and NaHCO_3_ were added at various concentrations to the PDA medium. The medium concentrations (w/v, g/mL) of 1, 4, 7 and 10% m/v were added to generate final salt stress. Likewise, alkali stress media (w/v, g/mL) of pH levels of 6.25, 6.50, 0.7 and 7.00 were obtained, with an untreated control of 5.77. To simulate a nutrient-deficient soil, PDA medium was diluted to 25, 50, 75 and 100%. To test for extreme temperatures, cultures were grown under 10, 20, 30 and 40 °C temperatures and growth rates measured. When NaCl^−^, pH and nutrient deficiency-amended media in the Petri dishes had solidified and cooled, a 5-mm-diameter mycelial disk of the respective test isolate was placed in the middle of the Petri dish and incubated at 25 ± 2 °C. For temperature tests, Petri dishes were incubated at different temperature levels. For each tolerance test, the culture conditions consisted of five replicates per treatment level and the radius of the colony was recorded using a ruler every 48 h for eight days.

### Fungal Isolates Biosafety Tests on Crop Seeds

Eight treatments of fungal BCA and untreated control, with five replications, were laid out in a completely randomized design (CRD) in vitro. Seeds of tomato (*Solanum lycopersicum* ‘Hotstuff’), swiss chard (*Beta vulgaris* subsp. *vulgaris* ‘Fordhock giant’), beetroot (*B*. *vulgaris* subsp. *vulgaris* ‘Detroit’), dry bean (*Phaseolus vulgaris* ‘PAN 148’), pumpkin (*Cucurbita moschata* L.), maize (*Zea mays* L.) and sorghum (*Sorghum bicolor* L.) were purchased at NWK store, Mahikeng, SA and were tested for fungal pathogenicity. Following a modified procedure by Coninck et al. [27], seeds were disinfected with 10% NaOCl solution for 5 min, washed three times for 5 min with sterile distilled water. The seeds were put on sterile paper towel and allowed to air dry for 24 h inside a laminar flow cabinet (FILTA-MATIX) before being coated and tested. To avoid aggregation formation, each fungal isolate was dissolved in water before being added to chitosan mixture and agitated frequently to make a slurry solution. Previously sterilized and dried seeds were submerged in their corresponding slurry solutions for 2–5 s before being allowed to air dry for 48 h at room temperature, i.e., 27 °C. Ten seeds per treatment were tested for germination in Petri dishes using Whatman No. 1 filter paper and 5 mL of sterile distilled water. When roots longer than 5 mm were seen, seeds were deemed to have germinated and inspected for necrosis and rotting. The experiment was repeated twice for validation, data compared and did not differ between the experiments.

## Data Analysis

Based on the homoscedascity assumption, i.e., that different fungal species vary in their tolerance to abiotic factors and may also differ in their biosafety on seed germination, the data were subjected to analysis of variance (regression) using SAS version 9.4 package (SAS Institute Inc., Cary, NC, USA) to test significance of the variation. Tukey’s Honestly Significant Difference (HSD) post-hoc test was used for mean comparisons at the probability level of 5%. Fungal dose–response curves were modeled by the regression estimations.

## Results

### Soil Characteristics

The results on soil physical properties indicated that the soils were silt loam (bulk density: 1 420 kg/m, field capacity: 320 mm/m, wilting point: 120 mm/m and porosity: 46%), slightly acidic with pH ranging from 5.85 to 6.25, EC ranged between 132.12 and 151.80 µs, while OC ranged from 22.50 to 26.10% and local temperatures fluctuated from 18 to 40 °C during collection of soil samples.

### Indigenous Fungal Isolates

Fungal identities were confirmed through molecular identification as follows: *A*. *flavus*, *A*. *terreus*, *Penicillium* sp. AL-38 IRH-2012b, *T*. *minioluteus*, *T*. *purpureogenus*, *T*. *sayulitensis*, *T*. *ghanense* and *T*. *viride* (Table 1).

**Table 1:** Molecular identification on indigenous fungal isolates

GenBank accession	Nearest BLAST match	Max. identity (%)
MT645322.1	*A*. *flavus*	100.00
MT316343.1	*A*. *terreus*	99.83
KC341982.1	*Penicillium* sp. AL-38 IRH-2012b	94.32
AB872818.1	*T*. *purpureogenus*	100.00
MN788118.1	*T*. *minioluteus*	97.69
MZ014549.1	*T*. *sayulitensis*	100.00
MF078652.1	*T*. *ghanense*	100.00
MW456070.1	*T*. *viride*	100.00

### Response of Indigenous Fungal Isolates to Extreme Environments

**Fig. 1:**
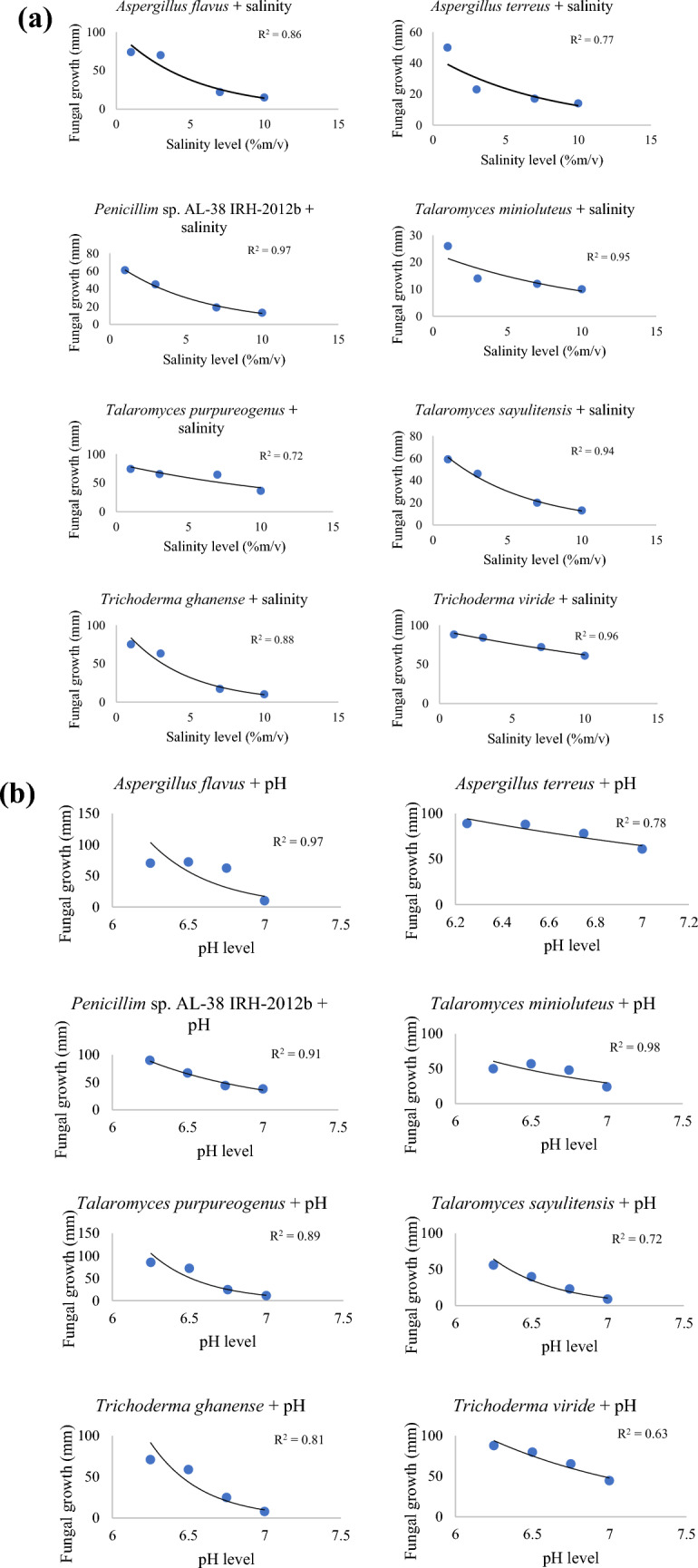

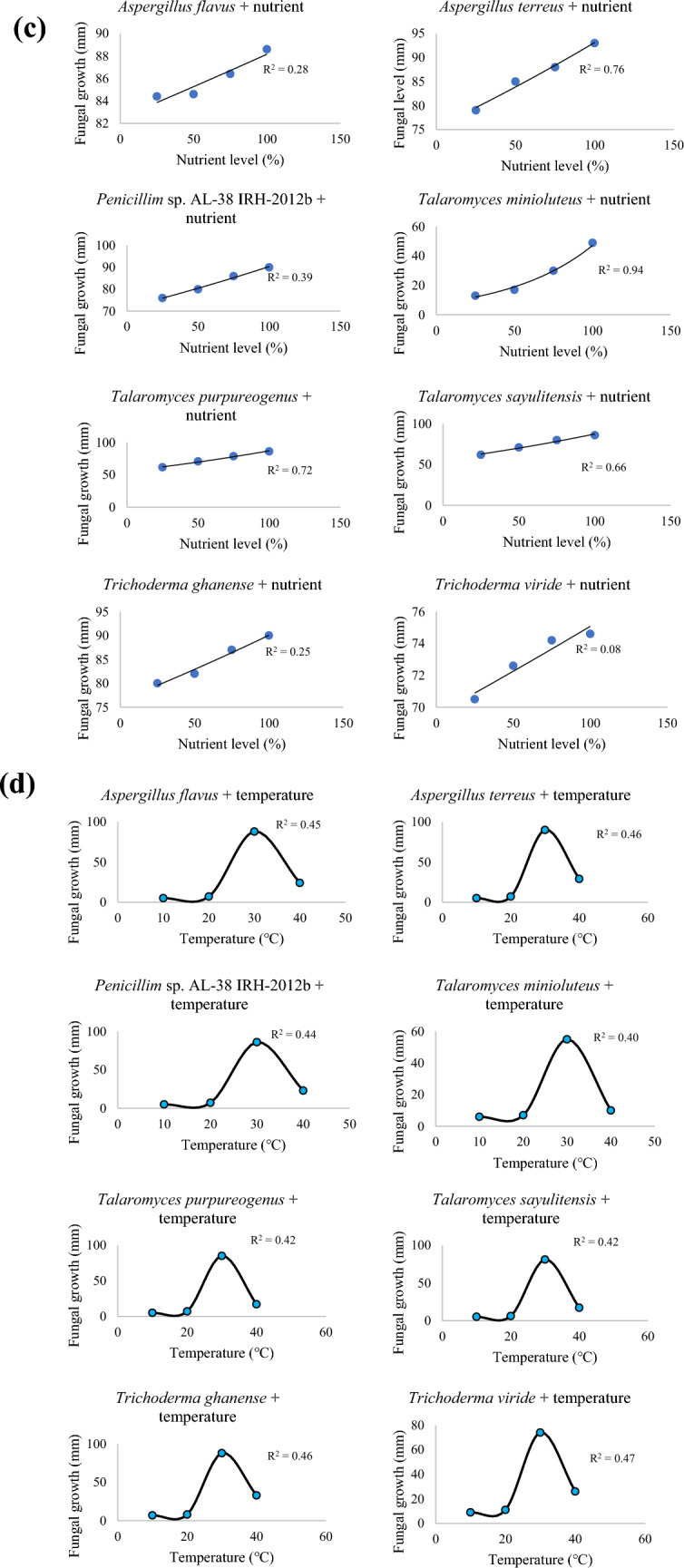
**a** Effect of different levels of salinity on fungi radial growth (mm) at eight days incubation period. **b** Effect of different levels of pH on fungi radial growth (mm) at eight days incubation period. **c** Effect of different levels of nutrient on fungi radial growth (mm) at eight days incubation period (n = 20). **d** Effect of different levels of temperature on fungi radial growth (mm) at eight days incubation period (n = 20)

The growth responses of *A*. *flavus*, *A*. *terreus*, *Penicillium* sp. AL-38 IRH-2012b, *T*. *minioluteus*, *T*. *purpureogenus*, *T*. *sayulitensis*, *T*. *ghanense* and *T*. *viride* differed significantly (*P* ≤ 0.05, F = 840.36, degrees of freedom = 7) to various levels of salinity, pH, nutrient deficiency and temperature (Fig. 1).

#### Salinity

**Fig. 2:**
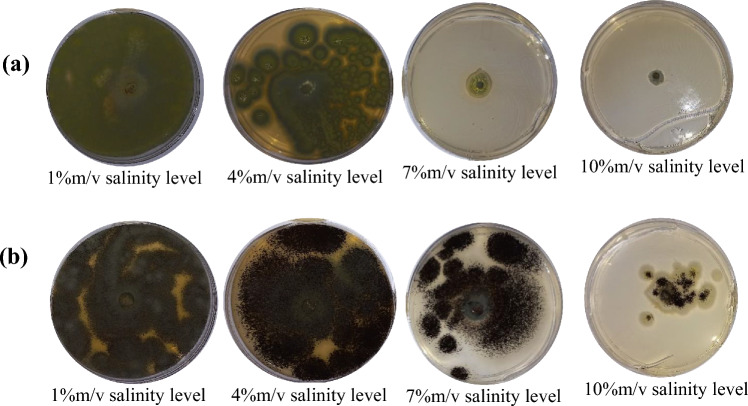
**a** *Aspergillus flavus* radii growth under salt amended PDA at 21 days after incubation. **b** *Talaromyces minioluteus* radii growth under salt amended PDA at 21 days after incubation

The isolates’ radii shrank significantly (*P* ≤ 0.05, F = 888.15, degrees of freedom = 7) with increasing salt concentration, except *T*. *viride* and *T*. *purpureogenus* that demonstrated superior adaptation (Table 2, supplementary data file). All eight selected fungal isolates thrived at 1%m/v salt concentration, with *T*. *sayulitensis* showing the least tolerance to salt stress than the other isolates. The radii of *A*. *flavus* significantly decreased as salt concentration increased (Fig. 2a). Nonetheless, all eight isolates could grow at 10% salt concentration (Fig. 2b).

#### PH

**Fig. 3:**
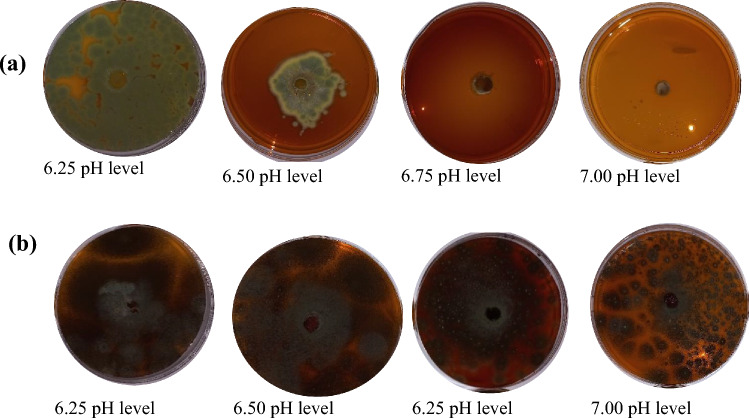
**a** *Talaromyces purpureogenus* radii growth under pH-amended PDA at 21 days after incubation. **b** *Trichoderma viride* radii growth under pH-amended PDA at 21 days after incubation

There was a highly significant difference (*P* ≤ 0.05, F = 687.86, degrees of freedom = 7) among all the eight fungal isolates at all levels (Table 3, supplementary data file). From the *Talaromyces* genus, *T*. *purpureogenus* adapted to changing pH levels (Fig. 3a) compared to *T*. *minioluteus* and *T*. *sayulitensis*. Even though *T*. *viride* showed better adaptation to pH levels (Fig. 3b), *T*. *ghanense* failed to grow at 6.75 pH-amended PDA. *Penicillium* radii growth decreased as pH levels increased but was still able to grow at a slow rate. Both *Aspergillus* species halted their radii growth at 1% pH level.

#### Nutrient Deficiency

**Fig. 4:**
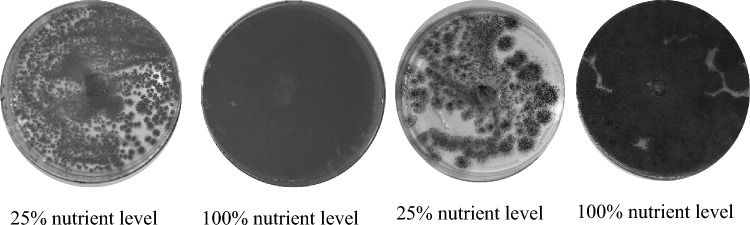
*Penicillium* sp. AL-38 IRH-2012b (left) and *Trichoderma viride* (right) radii growth under nutrient amended PDA at 21 days after incubation

Change in nutrient status had significant effect (*P* ≤ 0.05, F = 104.38, degrees of freedom = 7) on seven fungal isolates, except *T*. *minioluteus*. Even at 100% nutrient status, *T*. *minioluteus* showed the least growth rate (Table 4, supplementary data file). Although their thickness was reduced and was growing sparsely (Fig. 4). The results further showed that the *A*. *flavus* tolerated the nutrient stress better than the other isolates.

#### Temperature

At 10 and 40 °C, none of the fungal isolates examined grew. The optimal growth temperatures varied from 27 to 32 °C and did not differ significantly (*P* ≤ 0.05) between eight of the local isolates. The current study demonstrated that there was no growth observed on the isolates at temperatures below 10 °C. Additionally, increasing the temperature above 40 °C resulted in the inhibition of mycelial growth (Table 5, supplementary data file). However, it was observed that it kept the fungal spores viable. Isolates’ growth rates increased when the temperature was raised from above 20 °C. The range between 27 and 30 °C was found to be ideal for mycelial development.

### Fungal Isolates Effects on Crops’ Seeds

**Fig. 5:**
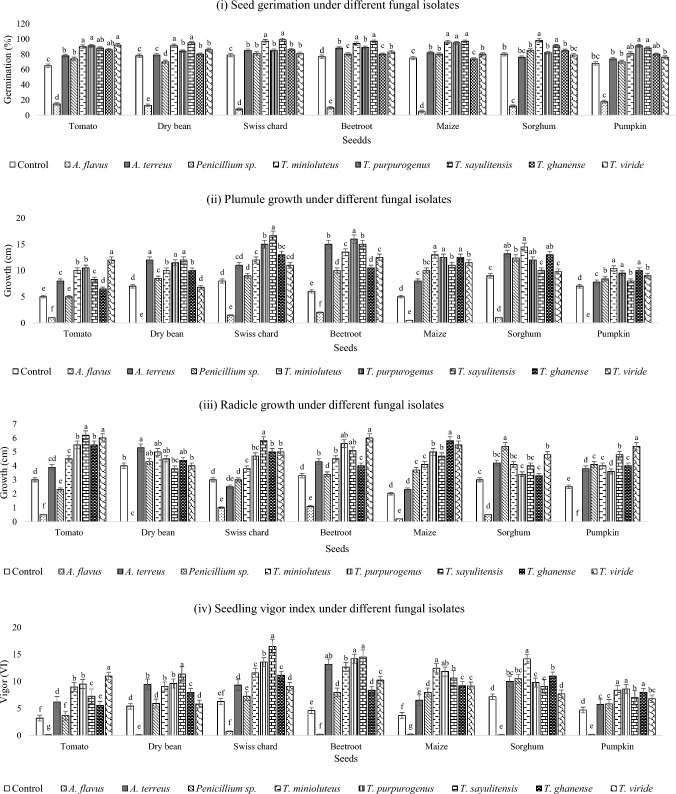
Response of (i) seed germination, (ii) radicle growth, (iii) plumule growth and (iv) seedling vigor index of different seeds at seven days exposure to different fungal isolates

Based on seed germination rate, plumule growth, radicle growth and seedling vigor index, the two *Trichoderma* species showed the highest seedling growth, followed by *Talaromyces*, *A*. *terreus* and *Penicillium* sp. AL-38 IRH-2012b, respectively. While *A*. *flavus* inhibited seed germination and caused seed rot. *Aspergillus terreus* promoted seed germination and subsequent improved seedling growth (Fig. 5).

## Discussion

Soil abiotic stress is a worldwide problem and has become a major factor limiting agricultural and forestry production [28]. Soil characteristics influence establishment and subsequent growth of micro-organisms in the rhizosphere. Due to their failure to colonize the host rhizosphere in stressful natural conditions, several fungal BCA that have promising potential when tested in vitro often fail to thrive under field conditions. As such, to maintain viability of BCA spores throughout storage and following formulation operations, tolerance to extreme conditions is important. The tolerance of plants to abiotic and biotic stress can be improved by the application of BCA [29]; however, the applied BCA isolates must acclimatize to the abiotic stress well.

### Fungal Isolates Tolerance to Salt

Results from the current study showed that the growth of *T*. *viride* was not hindered by 10% (1709.40 mM) NaCl. However, as incubation time increased, the promotion effect gradually weakened for the seven fungal isolates, except for *T*. *viride*, thus exhibiting remarkable NaCl tolerance. This observation showed that different fungal species from the same genus can have different tolerance capacity for the same salinity level, which could be attributed to different metabolites from individual species. For instance, Yusnawan et al. [30] identified high concentrations of isoprophyl-1-methyl, ledene oxide and acetone in *T*. *virens* T.v3. While gurjunene, butanol and himachalene were found in *T*. *asperellum* T.v8 [31]. This demonstrated the potential of *T*. *viride* to be applied in soils with high salinity levels.

This has been corroborated in previous field studies where *T*. *asperellum* RM-28, which tolerates saline (4% NaCl) environments, improved the growth and resistance of sorghum-sudan grass (*Sorghum bicolor* ssp. *drummondii*) seedlings [32]. Similarly, Zhang et al. [33] observed that Cucumber (*Cucumis sativus* ‘Chuanlv 21’) leaves' total chlorophyll content dropped from 1.29 to 1.00 mg/g when NaCl concentrations increased (0, 100 and 200 mM). Application of *T*. *atroviride* (HN082102.1), however, caused the content of total chlorophyll to rise by 16.57, 13.93 and 19.68%. All the tested indigenous fungal isolates have potential to be applied for crop improvement and protection in soils with approximately 4% (700 mM) salinity.

### Fungal Isolates Tolerance to pH

The pH of the rhizosphere affected the population of soil microbes as well as a number of metabolic activities. Also, the pH of the growth medium affected mycelial development and bio-control potential by regulating the synthesis of extracellular enzymes. The indigenous *T*. *viride* was more tolerant to a wide range of pH, followed by *T*. *purpureogenus* and then *Penicillium* sp. AL-38 IRH-2012b. This further demonstrated how different enzymes and other metabolites from different fungal isolates within the same genus play a role in their respective adaptation capabilities. These metabolites are known to aid in adapting to a wide range of pH and promote greater mycelial development and biological activity [34]. *Trichoderma viride*, *T*. *purpureogenus* and *Penicillium* sp. AL-38 IRH-2012b species’ potential as inoculant bio-agents in soils where changing pH is anticipated is supported by their improved tolerance and survival at increased pH levels.

The current results showed that *T*. *viride* changed the pH of the medium to levels close to neutrality, which is an impressive development that sets it apart from the other examined isolates. This was observed when the association between biomass production and pH modification in the pH media was evaluated. Given that the influence of pH stress reduces the generation of mycelium, there is sufficient evidence to support the use of *T*. *viride* in crop production under pH soils. This is due to the fact that not only did the isolate prove adaptation to high pH, but altered the pH to the level more suitable for most agricultural crops [35].

Cabral-Miramontes et al. [36] identified that *T*. *harzianum* has an adaptation to high pH, like *T*. *viride* isolates from the current study, it exhibited the ability to alter the pH of the medium. As a result, the effects of pH do not prevent mycelial development, and biomass increase is beneficial in the creation of the plant–microbe correlation since mycelium serves to house plant roots and give nutrients, resulting in a mutualistic connection. Extreme pH settings do not harm these micro-organisms. They adjust to their surroundings by secreting extracellular metabolites to reduce stress. These molecules work in conjunction with the proper molecular niche development for the advantageous exchange of protons and this is crucial for the adjustment of valences in chemical compounds. As a result, they are taken up by plants and microbes as macro- and micronutrients [37].

### Fungal Isolates Tolerance to Nutrient Deficiency

Survival BCA techniques involve competition for the growing site and eventual overtaking it. The capacity to develop quickly is the key advantage in the race for resources like nutrients and space [38]. Considering that there are possibilities of fungal BCA to grow slowly in a nutrient-poor environment, they might not be able to successfully establish in the growth site and outcompete other local plant-pathogenic micro-organisms when placed in such soils. Such BCA with weak growth potential may also not survive in the soil for a long period. Important bio-control techniques involve competition for the growing site and eventual overtaking it [39].

The fungal isolates morphology was examined under a compound microscope, and it was observed that the hyphae in the 100% nutrient level had distinct and turgid hyphal structures. When compared to *A*. *terreus* at equivalent test durations and nutrient levels, this isolate developed flaccid hyphae; the growth and development phenotype reverse of *Penicillium* sp. AL-38 IRH-2012b and *T*. *viride*. It could be that the metabolism reacts differently to prevent potential hydroxyl ion damage. Furthermore, it had a sparse structure, indicating that it does not cluster to react at excessive nutrient deficiency [36]. The observed thin and sparsely distributed growth (Fig. 4) could mean that under insufficient nutrient level, the BCA would be less effective and may be even overgrown by other pathogenic competing microbes.

### Fungal Isolates Tolerance to Temperature

Fungal BCA tolerance capacity to temperature fluctuations is an important criterion for the organism's survival under field conditions. In this study, all eight selected indigenous isolates showed the best growth at 27–30 °C. *Trichoderma* isolates were found to show comparatively better growth characteristics in all tested temperatures, i.e., from 10 to 40 °C. In contrast, it was noted that *Talaromyces* isolates exhibited the lowest growth level. Similarly, Affokpon et al. [38] observed that the sporulation as well as mycelia development were both greater at 28 °C. Following three weeks of the incubation, fungal isolates were able to develop normally and distinguished by strongly stimulated sporulation.

All the isolates failed to develop at temperatures from 10 °C and below, in contrast to *Trichoderma* species, albeit at significantly slower rates than the other temperatures examined. One of the species' survival mechanisms in extremely cold conditions, such those between 10 and 20 °C, seems to be the induction of chlamydospores. *Trichoderma* species spores demonstrated heat resistance at temperatures between 45 and 75 °C. Contrarily, three strains of *Trichoderma*, ID11D, ID4A and ID4B, were found to be able to maintain the viability of spores at 75 °C in this study heat tolerance test by Kucuk and Kivanc [40]. Current findings are comparable with those of Kucuk and Kivanc [40] which suggested that the fungal isolates were primarily adapted to local environmental conditions from when they were collected.

### Fungal Isolate Effects on Crops’ Seeds

While *A*. *flavus* caused seed rot and reduced seedling growth, *A*. *terreus* improved seed germination and seedling growth of tested vegetables. This shows that species from the same genus can inhibit seed germination and seedling growth, while other species promote growth. *Aspergillus* is a genus of filamentous fungi that are typically opportunistic, prevalent in soil, air and decaying plant matter [41]. Previous research has shown the inhibitory effects of Aflatoxin B1 from *A*. *flavus* on seed germination and seedling development in other agricultural plants [42]. Yoo et al. [43] previously reported that four weeks tomato seed treatment with *A*. *terreus* isolates, JF02, JF07, JF27 and JF44, seedlings had significantly increased shoot length in comparison with the control group.

In the present study, it was observed that *Penicillium* isolate promoted the growth of grain seeds, maize and sorghum, more than those of vegetable seeds. An increased root biomass could promote abiotic stress tolerance and aiding the plants in absorption of water and nutrients in far proximity. The stimulation of seed germination by *Penicillium* sp. AL-38 IRH-2012b may have resulted from synthesis of plant growth hormones such auxins, gibberellic acids, abscisic acid, ethylene and jasmonate. It has been shown in several investigations that *Penicillium* species generate gibberellic acid, which is necessary for the mitotic division of seeds [44]. In a study by Mushatq et al. [45], pre-soaking tomato seeds with *Penicillium* isolates considerably enhanced seed germination and subsequent seedling growth of up to 90% higher than untreated seeds.

The two *Trichoderma* species showed the fastest seed germination and seedling growth compared to other tested isolates. Furthermore, 100% seed germination was observed on *Trichoderma*-treated seeds. According to Shahid et al. [46], chickpea (*Cicer arietinum* L.) germination and vigor were improved when seeds were treated with *T*. *viride*; while Lalitha and Arunalakshmi [44] discovered improved seed germination and root length in mustard. The application of *T*. *viride* promoted wheat plant height, root length, leaf length, number of leaves and grain production [47]. *Trichoderma ghanense* improved shoot and root length of rye (*Secale cereale* L.) seedlings [48]. The inoculation of *Trichoderma* isolates led to an increase in auxin, a decrease in cytokinin and abscisic acid content in melon (*C*. *melo*) and further stimulated melon growth [49].

Compared to untreated control, *T*. *minioluteus* improved germination rate of *S*. *lycopersicum* significantly by 99%. *Talaromyces* isolates promoted the formation of lateral roots in vegetable seeds, which would result in an improved nutrient uptake capacity and an increase in the biomass of the roots and shoots. *Talaromyces* species have been shown to increase crop growth through their abilities to produce siderophores, Indole Acetic Acid (IAA) and dissolving phosphorus [50].

## Conclusion

The findings demonstrated that although being indigenous, *Aspergillus*, *T*. *sayulitensis* and *T*. *ghanense* did not thrive in conditions of high salinity and pH; *Penicillium* sp. AL-38 IRH-2012b failed to grow at lower nutrition levels and all fungal isolates failed to grow at temperatures between 10 and 20 °C. The production of chlamydospores by *Trichoderma* was acknowledged as a means of surviving unfavorable conditions. Additionally, *Aspergillus* species from the same genus can have both favorable and unfavorable effects on crops. The adaptation of BCA to habitats exposed to harsh and sudden fluctuating climatic conditions is a significant challenge to the transition from conventional to organic agriculture, even more so now due to climate change. The fact that the assessed fungal isolates live a multitrophic existence in nature; one that includes soil saprophytism and plant root endophytism indicates that they have the potential to adapt throughout time and geographical zones. The tolerance characteristics of fungal isolates indicate the need for continuous study of soil-borne fungal isolates with particular focus on the probability of certain species evolving into being plant parasitic.

### Supplementary Information

Below is the link to the electronic supplementary material.

Supplementary file 1 (DOCX 28 kb)
