# Supplementary material for: In Vitro Assessment of Eight Selected Indigenous Fungal Isolates Tolerance to Various Abiotic Stresses and their Effects on Seed Germination
Source: Curr Microbiol. 2023 Oct 24;80(12):386. doi: 10.1007/s00284-023-03507-6 (PMC10598106; doi:10.1007/s00284-023-03507-6)
Supplement: Supplementary file 1 — (DOCX 28 kb) [file 284_2023_3507_MOESM1_ESM.docx]

| Table 2 Fungal growth values at different doses of salinity-amended potato dextrose agar (PDA) media at 21 days after incubation (n = 40). | | | | | | | | | | |
| --- | --- | --- | --- | --- | --- | --- | --- | --- | --- | --- |
|  |  | Growth (mm) | | | | | | | | |
| Fungi |  | Control |  | 1%m/v |  | 4%m/v |  | 7%m/v |  | 10%m/v |
| *A. flavus* |  | 88.20±1.78^a^ |  | 89.80±2.86^b^ |  | 71.20±1.64^b^ |  | 21.80±0.84^c^ |  | 14.80±0.44^c^ |
| *A. terreus* |  | 89.80±2.48^a^ |  | 53.00±2.44^e^ |  | 49.40±15.02^c^ |  | 17.00±0.70^de^ |  | 12.80±0.83^d^ |
| *Penicillium* sp. |  | 88.20±1.48^a^ |  | 62.40±2.41^c^ |  | 43.40±2.30^c^ |  | 17.60±1.67^de^ |  | 12.20±0.83^d^ |
| *T. minioluteus* |  | 88.20±5.63^a^ |  | 84.60±1.81^b^ |  | 76.40±1.14^b^ |  | 63.80±1.92^b^ |  | 35.60±1.14^b^ |
| *T. purpureogenus* |  | 86.20±3.89^a^ |  | 58.60±1.14^d^ |  | 48.20±3.19^c^ |  | 20.80±1.48^c^ |  | 13.00±0.70^d^ |
| *T. sayulitensis* |  | 82.20±2.38^a^ |  | 26.60±1.34^f^ |  | 14.00±0.00^d^ |  | 12.20±0.44^f^ |  | 10.00±0.70^e^ |
| *T. ghanense* |  | 89.20±1.09^a^ |  | 75.40±2.30^b^ |  | 65.80±1.92^b^ |  | 15.80±0.83^e^ |  | 11.80±1.64^d^ |
| *T. viride* |  | 89.60±6.02^a^ |  | 74.40±0.89^b^ |  | 64.80±1.30^a^ |  | 55.40±1.67^a^ |  | 42.60±1.81^a^ |
| zColumn means followed by the same letter were not different according to Tukey’s HSD Post-hoc at the probability level of 5%, average ± standard deviation error. | | | | | | | | | | |

| Table 3 Fungal growth values at different doses of pH-amended potato dextrose agar (PDA) media at 21 days after incubation (n = 40). | | | | | | | | | | |
| --- | --- | --- | --- | --- | --- | --- | --- | --- | --- | --- |
|  |  | Growth (mm) | | | | | | | | |
| Fungi |  | Control (5.75) |  | 0.1% (6.25) |  | 0.4% (6.50) |  | 0.7% (6.75) |  | 1.0% (7.00) |
| *A. flavus* |  | 88.20±1.78^a^ |  | 70.40±2.30^c^ |  | 72.20±1.30^c^ |  | 62.40±2.30^b^ |  | 10.00±0.00^e^ |
| *A. terreus* |  | 89.90±2.48^a^ |  | 85.20±3.11^b^ |  | 71.80±1.30^c^ |  | 53.00±2.23^c^ |  | 62.20±1.64^b^ |
| *Penicillium* sp. |  | 90.00±0.00^a^ |  | 85.20±1.48^b^ |  | 69.20±1.92^c^ |  | 43.60±2.60^d^ |  | 36.60±1.67^c^ |
| *T. minioluteus* |  | 48.20±5.63^d^ |  | 51.40±1.14^d^ |  | 26.60±1.14^d^ |  | 10.40±1.81^d^ |  | 7.20±1.30^d^ |
| *T. purpureogenus* |  | 86.20±3.89^ab^ |  | 88.80±1.30^a^ |  | 53.60±4.39^b^ |  | 12±2.19^a^ |  | 8.40±4.15^e^ |
| *T. sayulitensis* |  | 82.20±2.38^b^ |  | 60.00±1.58^f^ |  | 37.60±1.94^e^ |  | 10.40±1.14^e^ |  | 7.00±0.70^e^ |
| *T. ghanense* |  | 89.20±1.09^a^ |  | 71.20±1.30^c^ |  | 70.60±1.10^c^ |  | 9.60±0.54^f^ |  | 10.00±0.00^e^ |
| *T. viride* |  | 74.60±6.02^c^ |  | 88.60±1.34^a^ |  | 81.60±1.14^b^ |  | 71.20±4.65^b^ |  | 79.80±178^a^ |
| zColumn means followed by the same letter were not different according to Tukey’s HSD Post-hoc at the probability level of 5%, average ± standard deviation error. | | | | | | | | | | |

| Table 4 Fungal growth values at different doses of nutrient-amended potato dextrose agar (PDA) media at 21 days after incubation (n = 40). | | | | | | | | | | |
| --- | --- | --- | --- | --- | --- | --- | --- | --- | --- | --- |
|  |  | Growth (mm) | | | | | | | | |
| Fungi |  | Control |  | 25% |  | 50% |  | 75% |  | 100% |
| *A. flavus* |  | 88.20±1.78^a^ |  | 84.40±2.70^a^ |  | 84.60±3.20^a^ |  | 86.40±3.04^a^ |  | 88.20±1.78^a^ |
| *A. terreus* |  | 89.80±2.48^a^ |  | 68.80±6.18^c^ |  | 84.00±8.21^a^ |  | 89.60±0.89^a^ |  | 89.80±2.48^a^ |
| *Penicillium* sp. |  | 88.20±1.48^a^ |  | 75.60±4.27^b^ |  | 82.20±1.92^a^ |  | 86.40±3.36^a^ |  | 88.20±1.45^a^ |
| *T. minioluteus* |  | 88.20±5.63^a^ |  | 14.20±1.30^e^ |  | 14.60±1.51^c^ |  | 24.60±4.61^d^ |  | 78.20±5.63^b^ |
| *T. purpureogenus* |  | 86.20±3.89^a^ |  | 61.60±6.06^d^ |  | 70.80±9.73^b^ |  | 78.80±3.70^b^ |  | 86.20±3.89^a^ |
| *T. sayulitensis* |  | 82.20±2.38^a^ |  | 68.00±6.44^c^ |  | 73.20±3.70^b^ |  | 82.60±3.74^a^ |  | 85.60±3.64^a^ |
| *T. ghanense* |  | 89.20±1.09^a^ |  | 85.40±3.13^a^ |  | 88.60±1.94^a^ |  | 88.00±3.08^a^ |  | 89.20±1.09^a^ |
| *T. viride* |  | 89.60±6.02^a^ |  | 70.00±6.44^bc^ |  | 74.20±4.43^b^ |  | 72.60±3.43^c^ |  | 84.60±6.02^a^ |
| zColumn means followed by the same letter were not different according to Tukey’s HSD Post-hoc at the probability level of 5%, average ± standard deviation error. | | | | | | | | | | |

| Table 5 Fungal growth values at different temperatures on potato dextrose agar (PDA) at 21 days after incubation (n = 40). | | | | | | | | | | |
| --- | --- | --- | --- | --- | --- | --- | --- | --- | --- | --- |
|  |  | Growth (mm) | | | | | | | | |
| Fungi |  | Control (27 ℃) |  | 10 ℃ |  | 20 ℃ |  | 30 ℃ |  | 40 ℃ |
| *A. flavus* |  | 88.20±1.78^a^ |  | 5.00±0.00^d^ |  | 7.40±0.54^cd^ |  | 82.40±0.85^a^ |  | 23.80±0.83^d^ |
| *A. terreus* |  | 89.80±2.48^a^ |  | 5.00±0.00^d^ |  | 7.00±0.00^d^ |  | 80.60±0.79^a^ |  | 29.60±1.51^b^ |
| *Penicillium* sp. |  | 88.20±1.48^a^ |  | 5.00±0.00^d^ |  | 7.60±0.54^c^ |  | 80.10±0.32^a^ |  | 22.00±0.70^e^ |
| *T. minioluteus* |  | 88.20±5.63^a^ |  | 5.80±0.44^c^ |  | 6.20±0.44^e^ |  | 68.20±0.71^c^ |  | 9.40±0.54^h^ |
| *T. purpureogenus* |  | 86.20±3.89^a^ |  | 5.20±0.44^d^ |  | 6.20±0.44^e^ |  | 78.10±0.87^b^ |  | 17.20±0.83^f^ |
| *T. sayulitensis* |  | 82.20±2.38^a^ |  | 5.00±0.00^d^ |  | 6.20±0.44^e^ |  | 76.30±0.46^b^ |  | 15.80±2.38^g^ |
| *T. ghanense* |  | 89.20±1.09^a^ |  | 6.60±0.54^b^ |  | 8.20±0.11^b^ |  | 82.20±0.54^a^ |  | 32.00±0.70^a^ |
| *T. viride* |  | 89.60±6.02^a^ |  | 8.80±0.72^a^ |  | 12.00±0.70^a^ |  | 74.60±1.05^b^ |  | 26.60±1.14^c^ |
| zColumn means followed by the same letter were not different according to Tukey’s HSD Post-hoc at the probability level of 5%, average ± standard deviation error. | | | | | | | | | | |

| Table 6 Analysis of variance (ANOVA) for fungal growth under different conditions *in vitro* at 21 days after incubation (n = 40). | | | | | |
| --- | --- | --- | --- | --- | --- |
| Salinity | | | | | |
| Source | Degrees of freedom | Sum of squares | Mean square | F | P |
| Replication | 4 | 4.10000 | 1.02500 |  |  |
| Treatment | 7 | 12382.37500 | 1768.91071 | 888.15 | 0.0303 |
| Error | 28 | 35.50000 | 1.26786 |  |  |
| Total | 39 | 12421.97500 |  |  |  |
| pH | | | | | |
| Replication | 4 | 14.85000 | 3.71250 |  |  |
| Treatment | 7 | 26454.30000 | 3779.18571 | 687.86 | 0.0409 |
| Error | 28 | 97.95000 | 3.49821 |  |  |
| Total | 39 | 26567.10000 |  |  |  |
| Nutrient | | | | | |
| Replication | 4 | 65.150000 | 16.287500 |  |  |
| Treatment | 7 | 6966.975000 | 995.282143 | 104.38 | 0.0281 |
| Error | 28 | 341.650000 | 12.201786 |  |  |
| Total | 39 | 7373.775000 |  |  |  |
| Temperature | | | | | |
| Replication | 4 | 1.650000 | 0.412500 |  |  |
| Treatment | 7 | 2011.900000 | 287.414286 | 168.88 | 0.0282 |
| Error | 28 | 30.350000 | 1.083929 |  |  |
| Total | 39 | 2043.900000 |  |  |  |
